# Sustainable removal of azo dyes from real effluents using a biomass-derived composite

**DOI:** 10.1038/s41598-025-32434-8

**Published:** 2026-01-14

**Authors:** F. M. Mohamed, A. M. Alfalos, M. F. Alrakshy, M. A. Aborziza, K. A. Alfalous, Mohamed Abdel Rafea, Magdi E. A. Zaki, Mohamed R. El-Aassar, Mahmoud A. Roshdy

**Affiliations:** 1 Faculty of Earth Sciences, Beni-Suef University, P.O. 62521, Beni-Suef, Egypt; 2 Faculty of science, Alasmarya Islamic University, Zliten, Libya; 3 Department of Physics, College of Science, Imam Mohammad Ibn Saud Islamic University (IMSIU), 11623 Riyadh, Saudi Arabia; 4 Department of Chemistry, College of Science, Imam Mohammad Ibn Saud Islamic University (IMSIU), 11623 Riyadh, Saudi Arabia; 5 Department of Chemistry, College of Science, Jouf University, 2014 Sakaka, Saudi Arabia

**Keywords:** Seaweed, Anthracite, Chitosan composite material characterization, MO sorption, Chemistry, Environmental sciences, Materials science

## Abstract

In this study, the composite of Seaweed as biomass and Anthracite @ Chitosan (MS-An@Cs) was successfully prepared for the removal of methyl orange as one of toxic azo dye from real and aqueous solutions. The physicochemical characteristics were investigated using X-ray diffraction, Fourier transform infrared spectroscopy, and thermogravimetric analysis. The effects of contact time, pH, adsorbent mass, agitation rate, initial dye concentration, and temperature were assessed through batch adsorption experiments, which revealed that the monolayer coverage was 38.2 mg/g. Moreover, several intervening diffusion types, such as intra-particle diffusion, were associated with the regulating phase in the MO sorption process. The MO equilibrium data was also examined using the linear versions of the Freundlich, DR, and Langmuir isotherms; the results indicated that the Langmuir fit the data best, with an R^2^ value of 0.92. Mo sorption kinetics onto MS-An@Cs were described by a pseudo-second-order model, and the linear and nonlinear regression analyses’ results matched well (R^2^ > 0. 0.965). It was proven by the thermodynamic parameters ΔH°, ΔS°, and ΔG° that the MO sorption process at 288–328 K was spontaneous and endothermic.the removal percentage of MO from real sample reached 97.5%. It was shown that the composite could be recycled at least six times after its recyclability and regeneration were examined. Additionally, adsorption kinetics and thermodynamics were examined. The created composite is therefore thought to be a more effective adsorbent that can effectively and economically treat industrial wastewater in Egypt. Batch scale-up studies reveal that just 53.72 g of the MS-An@Cs nanocomposite can effectively remove 95% of MO dye from 50 L of contaminated water at the tested concentration, while cost analysis estimates a low production cost of $0.0174 per gram, demonstrating its superior cost efficiency over conventional water purification methods reported in prior this study.

## Introduction

 The exploration of materials that have a harmful impact on living organisms is an essential domain of research. The adverse consequences of urban development and industrialization on human health and environmental conditions are becoming more pronounced each day. Numerous industries and production facilities release untreated waste directly into the ecosystem without undergoing proper waste treatment processes. Potential strategies to mitigate the impact of this issue on societal well-being and the environment are gaining increasing importance. It is documented that minimal levels of chemicals, particularly colorants used in industry, demonstrate potent contaminating characteristics. A vast number of colorants are produced annually for global use in significant volumes^1^. Among the key contaminants, synthetic colorants hold particular significance. Methyl Orange (MO) is considered a synthetic colorant because it possesses an azo group in its molecular framework^2^. The preference for synthetic azo dyes over natural dyes is driven by their cost-effectiveness and ease of production. A considerable proportion of commercially utilized synthetic dyes consists of azo dyes that exhibit toxicity, carcinogenicity, and mutagenicity^3^. Certain azo dyes may be toxic even without dissolving in aromatic amines; however, the toxicity of some azo dyes is relies on the production of compounds such as benzidine. In their 1992 study, Chung and Cerniglia proposed that benzidine, as well as phenylene diamine (p-PDA) are carcinogenic constituents of azo dyes^4^. Benzidine has been shown to induce tumors in a range of human and animal models. Furthermore, phenylene diamine, which is also an azo dye constituent, is recognized as an allergenic substance. Reports indicate that a significant number of azo dyes and their metabolites can influence human health, causing allergic responses and other medical issues^3^. Azo dyes are frequently utilized without regard for their toxicological implications. The N = N–bond, which is resistant to dissolution, contributes to their toxic nature (IUPAC, 997). Various treatment techniques have been applied to safeguard water resources from toxic elements, yet additional studies are being conducted to improve their effectiveness. The resolution of this problem should align with sustainable practices^1^. To address the removal of colorants from wastewater, diverse physical-chemical and biological techniques have been used, such as coagulation^5,6^, adsorption^7,8^, photodegradation^9^, chemical oxidation^10^, flocculation^11^, electrodialysis^12^, and membrane filtration^13–15^. Of the various techniques, adsorption has proven to be highly effective, benefiting from the availability of different waste-derived substances. The incorporation of natural materials is significant for the economic viability and sustainability of the process^16,17^. Adsorption involves the migration of atoms, ions, or molecules in a solution onto the surface of an adsorbent, primarily taking place on the adsorbent’s surface phase^18^. The core principle of adsorption depends on the solubility tendencies of the substance and its attraction toward the solid phase. A dynamic equilibrium is achieved between the solid and liquid phase concentrations as the substance adsorbs onto the solid surface from the solution in a liquid-solid system. This equilibrium is critical for optimizing the adsorption efficiency between the levels of the substance in the aqueous and solid mediums^19^. All solids inherently have adsorption attributes, though their efficiency may differ. Numerous agricultural byproducts^20–27^ have been effectively used as sorbents and have undergone extensive evaluation to assess their effectiveness in addressing dye contamination. Among the key biowastes, *Posidonia oceanica* (PO) stands out as an endemic seagrass species, predominantly found in sandy coastal areas that span approximately 50,000 km² across the Mediterranean Sea^28^. Recent research reveals that seagrass (SG)-derived products, such as raw materials and activated carbon, show potential in pollutant adsorption. Activated carbon from SG has been used in various studies to extract both organic and inorganic pollutants from wastewater. This material is also capable of adsorbing pharmaceuticals that may negatively impact on the environment and human health, as well as assisting in water purification. Anthracite (An), recognized for its large surface area and mesoporous properties, has been studied as an effective adsorbent. This low-cost natural resource has been employed as a filtration medium since 1933^29^. To enhance the efficacy of seagrass (SG) raw material as a model adsorbent for methyl orange (MO) removal, combining anthracite (An) with the SG matrix to form a bio-nanocomposite is seen as an important strategy.

The optimization of critical adsorption parameters—such as pH of the dye solution, adsorbent dosage, and contact duration—was conducted using Response Surface Methodology (RSM) in conjunction with a Box-Behnken Design (BBD). RSM integrates advanced mathematical and statistical approaches to analyze the influence of independent variables, their interactive effects, and the systematic optimization of the entire process. A key advantage of the RSM-BBD approach lies in its ability to circumvent extreme experimental conditions while significantly minimizing the total number of trials required^30–32^.

Thus, the primary objectives of the present study are to (1) Impregnate anthracite onto the surface of Sea grass derived-activated carbon (MS) in the presence of chitosan for the effective of MO uptake. (2) Characterize the structural composition of MS-An@Cs composite, (3) Optimize the experimental parameters of temperature, pH, MO initial levels, amount of adsorbent, agitation period, and agitation speed, (4) Examine the adsorption isotherm models (Langmuir, Freundlich, Temkin and DR), and kinetics (Pseudo first order (PFO), second order model (PSO), and intraparticle diffusion (IPD), (5) Assess the proposed adsorption mechanism.

## Materials and methods

### Materials

MO identified by its chemical structure as 4-[[(4-Dimethylamino) phenyl] azo] benzene sulfonic acid sodium salt (C.I. 13025, MW = 327.34 g/mol, 85% dye purity), was procured from a chemical laboratory. To prepare the desired concentration, the dye was diluted using deionized water. Chemicals such as sulfuric acid (H₂SO₄) and sodium hydroxide (NaOH) were employed to regulate the initial pH of the solution and were obtained from the same source. Raw anthracite (An) and chitosan (Cs) materials were supplied by the Holding Company for Water and Wastewater.

### Materials Preparation

#### Preparation of modified seaweed (MS)

**Scheme 1:**
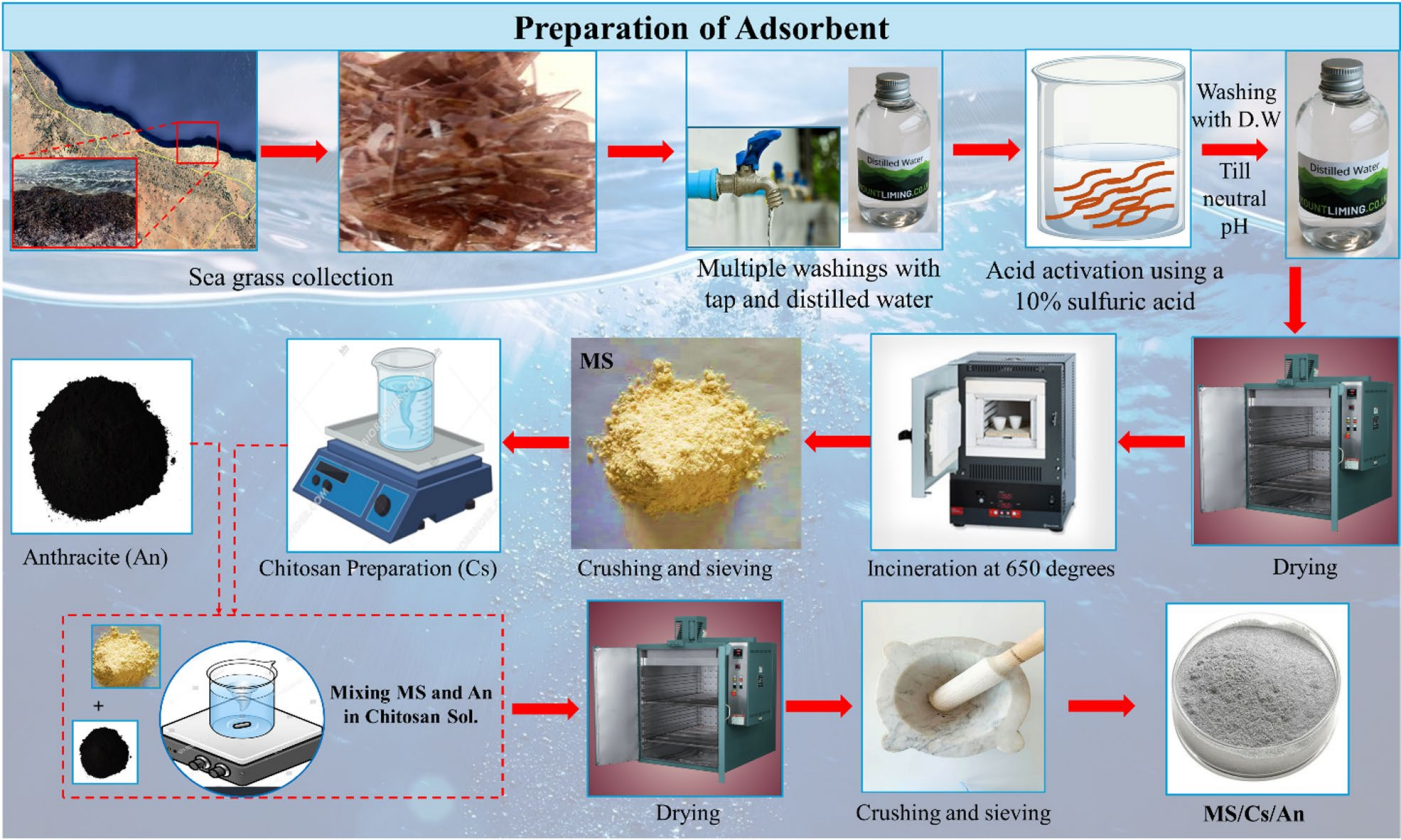
Experimental flowchart.

Seaweed (S) was collected from the coastal region of Zliten, Libya (Scheme 1). The seagrass was identified as *Posidonia oceanica*. It underwent multiple washings with tap water, followed by rinsing with distilled water, and was then air-dried at room temperature. Subsequently, the seaweed was cut into irregularly shaped particles ranging from 5 to 10 mm in size, filtered, and dried in an oven at 105 °C for 2 h. The dry sample was activated using a 10% sulfuric acid (H₂SO₄) solution for 24 h, maintaining a solid-to-liquid ratio of 1:6, to enhance the adsorption capacity of the adsorbent material. The samples were then washed with distilled water several times until the pH of the washing water reached neutrality, dried again in an oven at 105 °C for 3 h, and subsequently incinerated in a furnace at 650 °C for 2 h. The residue was crushed and powdered into fine particles by using an agate mortar and then sieved through a standard 75 μm mesh sieve to obtain a fine powder. Finally, the resulting material was stored in a glass bottle and take notation (MS) for use in adsorption experiment pretreatment.

#### Preparation of MS-An@Cs composite

Initially, 0.5 g of chitosan was combined with 100 ml of acetic acid at a concentration of 2% and stirred on a magnetic stirrer at 80 °C for 2 h, resulting in a high-viscosity solution. Subsequently, 2 g of the material (MS) was uniformly mixed with 0.5 g of natural Anthracite (An), maintaining a solid-to-solid ratio of 4:1. This mixture was then dissolved in 10 ml of the chitosan solution, stirred, and heated at 70 °C for 24 h. The minimal quantity of Anthracite was chosen to achieve the desired outcome while minimizing costs. The resulting mixture was gently ground and stored for future use, designated as the MS-An@Cs composite (Scheme 1).

### Materials characterization

A multitude of analytical techniques such as FTIR and X-ray diffraction performed on a Philips APD-3720 diffractometer, were used to characterize the materials MS and the MS-An@Cs composite. We used scanning electron microscopy (SEM, JSM-6700 F, JEOL, Japan) to analyze the morphological features. Furthermore, TGA (Q500 Thermogravimetric Analyzer) and DSC (TA Instruments Q2000 DSC) were carried out. Nitrogen adsorption/desorption at 77 K was used to calculate the specific surface area (SBET) using a gas sorption analyzer (Quantachrome, NOVA, version 11.04, USA). The amount of nitrogen gas adsorbed at the relative pressure (P/P0) was used to estimate the total pore volume (Vtotal). Using a surface area analyzer (Tristar II analyzer), the surface area, pore volume, and pore size of the MS and the MS-An@Cs composite were measured following an hour of degassing at 200 °C. The pore volume, pore size, and BET surface area (SBET) for MS and the MS-An@Cs composite were estimated using the Brunauer–Emmett–Teller (BET) and Barrett–Joyner–Halenda (BJH) equations, respectively.

#### Batches of adsorption experiment

.

This research utilized the batch adsorption method, where initial standard solutions of MO were created at a concentration of 500 µg/ml. Serial dilutions of MO were then prepared to establish an optimal calibration curve. In this investigation, various factors affecting the adsorption process were examined, including the dosage of MS-An@Cs, pH levels, concentrations of MO, temperature, agitation speed, and the duration of agitation between the adsorbent and the pollutant (Table 1). Six Falcon tubes (25 ml) were filled evenly with 35 mg of the prepared MS-An@Cs composite in order to examine the impact of pH. A 50 mg/L (50 ppm) solution of the adsorbate was added to the tubes. Next, either 0.1 N NaOH or 0.1 N H₂SO₄ was used to bring the pH of the solutions within a range of 2–9. The Falcon tubes were set up on an orbital compact shaker (Edmund Buhler, Germany) and rotated at 200 rpm for two hours.

Afterward, the effect of varying adsorbent mass s was analyzed by adding distinct amounts into another group of seven Falcon tubes, which were adjusted to the previously identified optimum pH. Each mass was tested in triplicate, with average results recorded for each specific amount. The examined mass ranged from 5 to 50 mg. Additionally, the impact of MO concentrations was explored within a range of 20–80 µg/ml, using the optimal conditions derived from previous steps. The affecting factors can be summarized in Table 1. MO was quantified using a UV-Vis spectrophotometer at a wavelength of 468 nm. The adsorption process was further clarified through investigations into adsorption isotherms, kinetics and thermodynamics. The error function was utilized to select the most suitable adsorption model, which was calculated accordingly.

**Table 1:** Affecting factors of MO onto MS-An@Cs composite.

Investigated parameter	Conditions								The other parameters
pH	2	3^a^	4	5	7	9			50 ppm of MO initial conc., 35 mg mass, 200 rpm and time 2 h
Mass (mg)	5	10	20	30	35^a^	40	50		pH (3.0), 50 ppm of MO initial conc., 200 rpm and time 2 h
Agitation time (min)	5	10	15	30	60	90	120^a^	150	pH (3.0), 50 ppm of MO initial conc., 35 mg mass, 200 rpm.
MO initial conc., (ppm)	20	30	40	50^a^	60	70	80		pH (3.0), 35 mg mass, 200 rpm, 200 rpm, and time 2 h
Agitation speed (rpm)	50	100	150	200^a^	250				pH (3.0), 50 ppm of MO initial conc., 35 mg mass, and time 2 h.
Temp (K)	293^a^	303	313	323	333				pH (3.0), 50 ppm of MO initial conc., 35 mg mass, 200 rpm, 200 rpm, and time 2 h

In our research, we investigated the regeneration of adsorbents using a 50 µg/ml concentration of MO-loaded adsorbent combined with HCl of 0.1 mol/L and was subsequently washed three times in 50 mL of ethanol 50% for 60 min. The ethanol was prepared at a 50% v/v concentration in total volume of 60 mL. Desorption studies was carried out by stirring and applying centrifugation to the used adsorbent for 90 min at 200 rpm at 30 °C. Multiple cycles of adsorption and desorption were conducted to evaluate the efficiency of MO removal by the regenerated adsorbent. The percentages of removal and the uptake capacity (mg/g) were estimated using Eqs. (1) and (2), where C0 (mg/L) indicates the initial concentration of MO, Ce (mg/L) is the equilibrium concentration in the solution, V represents the volume in liters, and M is the mass of the adsorbent in grams.

1 $${\text{Removal }}\% =\left( {{\mathrm{C}}0 - {\mathrm{Ce}}} \right)/{\mathrm{C}}0 \times {\mathrm{1}}00$$

2 $${\mathrm{qe}}=\left( {{{\mathrm{C}}_0} - {\mathrm{Ce}}} \right)*{\mathrm{V}}/{\mathrm{M}}$$

### BBD model

The BBD-RSM offers a robust and efficient experimental framework for optimizing the multivariable adsorption system of MO using MS-An@Cs. This approach significantly reduces the required number of tests while ensuring comprehensive analysis. In the present investigation, the BBD was employed to methodically evaluate the influence of three critical parameters—pH, adsorbent dosage (MS-An@Cs), and contact time on MO removal efficiency. The experimental design and subsequent data analysis were conducted using Design Expert 12.0 (Stat-Ease, Inc.), ensuring rigorous statistical validation.

Table 2 presents the defined experimental domain and the associated constraints for MO removal efficiency using the MS-An@Cs adsorbent. A quadratic polynomial model (Eq. (3)) was employed to quantitatively assess the impact of key adsorption parameters on the elimination of MO.

3 $$\:\mathrm{Y}=\:{{\upbeta\:}}_{0}+{\sum\:}_{\mathrm{j}=1}^{\mathrm{k}}{{\upbeta\:}}_{\mathrm{j}}{\mathrm{x}}_{\mathrm{j}}+{\sum\:}_{\mathrm{j}=1}^{\mathrm{k}}{{\upbeta\:}}_{\mathrm{j}\mathrm{j}}{\mathrm{x}}_{\mathrm{j}}+\sum\:_{\mathrm{i}}{\sum\:}_{<\mathrm{j}=2}^{\mathrm{k}}{{\upbeta\:}}_{\mathrm{i}\mathrm{j}}{\mathrm{x}}_{\mathrm{i}}{\mathrm{x}}_{\mathrm{j}}+{\mathrm{e}}_{\mathrm{i}}$$

In this model, Y denotes the removal efficiency of MO as the response variable, while x_i_ and xⱼ represent the independent variables (where *i* and *j* range from 1 to *k*). The constant term is given by β₀, whereas β_i_, βⱼⱼ, and β_i_ⱼ correspond to the regression coefficients for linear, quadratic, and interaction effects, respectively. Here, *k* signifies the total number of experimental factors, and *e* accounts for residual error in the model.

Table 3 presents the BBD matrix encompassing the three investigated variables along with their corresponding MO removal efficiencies. The adsorption experiments were conducted by introducing 25 mL of MO aqueous solution (50 mg/L concentration) along with a precisely measured quantity of MS-An@Cs adsorbent into a 50 mL conical centrifuge tube. The mixture was then subjected to continuous agitation at 200 r.p.m. for the predetermined contact duration using an orbital mechanical shaker.

**Table 2:** Codes and actual variables and their levels in BBD.

Code	Variables	−1	0	+ 1
A	pH	2	5.5	9
B	Mass (mg)	5	27.5	50
C	Contact time (min)	5	77.5	150

The MS-An@Cs adsorbent was separated from the aqueous phase through vacuum filtration utilizing a 0.45 μm membrane filter.

**Table 3:** Experimental matrix based on BBD approach for designing experiments and the corresponding response (MO removal).

Run	A: pH	B: mass (mg)	C: Time (min)	MO removal (%)
1	9	27.5	150	86.78
2	5.5	5	150	60.21
3	2	27.5	150	98.51
4	5.5	27.5	77.5	73.86
5	2	5	77.5	90.57
6	5.5	27.5	77.5	70.31
7	5.5	27.5	77.5	72.45
8	5.5	5	5	55.78
9	9	5	77.5	53.42
10	9	27.5	5	42.214
11	5.5	50	5	67.87
12	5.5	50	150	95.72
13	5.5	27.5	77.5	73.45
14	5.5	27.5	77.5	71.79
15	9	50	77.5	91.32
16	2	50	77.5	100
17	2	27.5	5	92.64

Subsequent spectrophotometric analysis of the filtrate was performed at the characteristic absorption wavelength (λmax = 520 nm) using UV-Vis spectroscopy to quantify the residual MO concentration. The decolorization efficiency was then determined through balance (Eq. 1).

## Result and discussion

### Characterization of MS and the MS-An@Cs composite

**Fig. 1:**
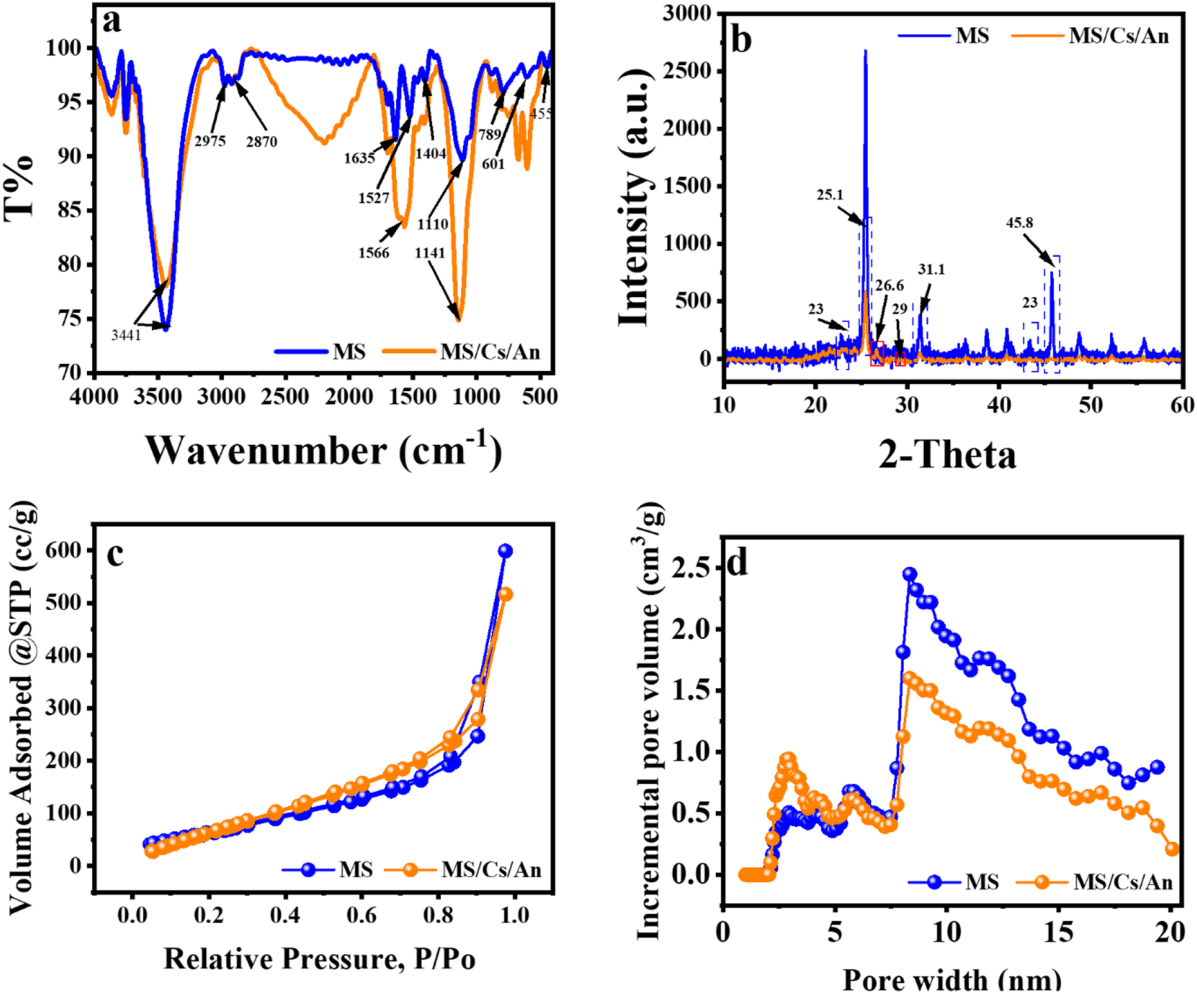
FTIR spectra (**a**), XRD patterns (**b**) N2 adsorption-desorption isotherm curves (**c**), and pore size distribution curve (**d**) of MS and MS-An@Cs.

Infrared spectral data for MS and the MS-An@Cs composite were collected across the wavenumber range of 400–4000 cm^−1^ (Fig. 1a). The spectra of all biomaterials were comparable. Understanding the adsorption mechanism requires knowledge of the functional groups present on the MS bio-adsorbent surface. In the MS IR spectrum, the key absorption peaks are attributed to cellulose, hemicellulose, and lignin, which are identifiable by the carbonyl, hydroxyl, and methoxy functional groups they contain. A broad absorption peak in the 3700–3100 cm^−1^ range, centered at 3441 cm^−1^, demonstrates the presence of –OH and –NH vibrational modes^33^. The bands at 2975 and 2870 cm, which are relatively small, are attributed to the asymmetric and symmetrical stretching vibrations of C–H in alkyl groups, respectively. At 1641 cm^−1^, the band is attributed to the amide C(O)NH_2_ bond, and the band at 1404 cm^−1^ is associated with the deformation vibration of –C–OH, coupled with the symmetrical O–C–O stretching vibration of the carboxylate group^34^. Notably, the band at 1404 cm^−1^ is identified as the crystalline band. Additional bands near 1527 cm⁻¹ is attributed to the stretching vibrations of C=C in aromatic carbon rings. The 1019 cm^−1^ band is linked to the presence of alcoholic groups^35^. Finally, all the other lower in frequency adsorption bands that exist on MS spectrum in the region 1000–400 cm^−1^, are assigned to calcite, dolomite and quartz crystalline phases that naturally exist in MS biomass^8,36,37^. In contrast, the infrared spectrum of MS-An@Cs displays features typical of carbon-based materials. The bands observed are similar to those in the MS spectrum, with the band at 1566 cm⁻¹ attributed to the C=C stretching vibrations in aromatic rings. This peak is more pronounced in MS-An@Cs, suggesting the inclusion of anthracite within the MS structure. Additionally, the weak shoulder at 1430 cm^−1^ is ascribed with the deformation vibration of –C–OH, coupled with the symmetrical O–C–O stretching vibration of the carboxylate group present in MS, as previously discussed. Finally, all the other lower in frequency adsorption bands that exist on MS-An@Cs spectrum in the region 1000–400 cm^−1^, are assigned to calcite, dolomite and quartz crystalline phases.

The MS and the MS-An@Cs were also characterized by XRD analyses as depicted by (Fig. 1b). In particular, the XRD pattern highlights the intermediate behavior of *Posidonia oceanica* sea plant between lignocellulosic and algal biomass. In particular, for both materials (MS and the MS-An@Cs) we observed the faint reflections centered near 2θ = 23° and 43° (corresponding to the analogous (002) and (100) planes of graphite). The observed peaks in graphitic carbon result from the stacking of graphene layers (002) planes and the in-plane crystalline structure of graphitic material (100) planes)^38^. In addition, the XRD spectrum of *Posidonia oceanica* shows a peak of high intensity at 25.4° corresponding to the quartz phase (SiO₂), a typical component of marine siliceous biomasses^38^. Moreover, sharp diffraction peaks at 2θ = 31.4° and 45.7° confirm the occurrence of dolomite [MgCa(CO₃)₂, JCPDS file No. 79-1346] and periclase [MgO, COD 96-901-3227] as inorganic impurities.

These phases may have existed beforehand or emerged during pyrolysis because of natural calcium, magnesium, and silicon impurities, or related phases associated with the *Posidonia oceanica* seagrass. In the spectrum of MS-An@Cs we observe a new sharp peak at 2θ = 26.1° corresponds to the amorphous graphite phase, which linked to the incorporation of anthracite within the MS structure^39^.

To explore the textural attributes of MS and MS-An@Cs, focusing on parameters such as pore size, pore volume, and BET surface area, nitrogen adsorption–desorption isotherms were utilized. Surface area analysis was performed through the application of the BET equation^40^, total pore volume was determined by transforming the adsorption volume at a relative pressure of 0.95 into the associated adsorbate volume^41^. Figure 1c displays the adsorption-desorption isothermal curves of nitrogen alongside the pore size distribution curve of MS and MS-An@Cs materials. The presence of type I and type IV isotherm profiles in both MS and MS-An@Cs, as classified by IUPAC, suggests that the samples possess both micro- and mesoporous structures. It should be emphasized that Type I isotherms are commonly characteristic of microporous materials with limited external surface areas. Nonetheless, the presence of mesopores in both MS and MS-An@Cs is indicated by the rise in nitrogen adsorption observed at elevated relative pressures. This complex pore arrangement contributes to MS’s elevated total pore volume of 0.928484 cm³/g and an average pore size of 7.57641 nm (Table 4), making it well-suited for the adsorption of larger molecules. Conversely, the MS-An@Cs has a reduced total pore volume of 0.288 cm³/g, and the average pore diameter of 1.36 nm than MS, which may be attributed to the partial pore obstruction by chitosan (Cs).In summary, the distinct pore and surface area properties of both AC(C) and AC(H) indicate their potential adaptability for diverse adsorption applications.

Hysteresis loops H4 was observed in the nitrogen adsorption isotherms between P/P₀ = 0.1 and 1.0 correspond to type-IV isotherms, confirming the mesoporosity of the materials. The dV/d(logD) metric serves as a tool for analyzing partial porosity in relation to various ranges of pore diameters. Table 4 summarizes the textural features of the synthetic adsorbents in detail. According to Table 4, the pore diameters of all the prepared materials are within the mesoporous range (2 nm < pore size < 50 nm). According to BET analysis, the surface areas of MS and MS-An@Cs were 245.09 and 314.54, respectively, with corresponding pore sizes of 7.57641, and 5.09314 nm Table 4. The observed increase in the surface area of the MS-An@Cs composite is attributed to the integration of anthracite. On the other hand, compared to MS, MS-An@Cs composite exhibited reduced pore volumes and average pore diameters, attributed to the partial pore obstruction by chitosan (Cs).

**Table 4:** Textural parameters of MS and MS-An@Cs were obtained from the N2 adsorption/desorption isotherms.

Sample	BET Surface Area (m²/g)	Total Pore Volume (cm³/g)	Average Pore Diameter (nm)
MS	245.09	0.928484	7.57641
MS-An@Cs	314.54	0.800998	5.09314

**Fig. 2:**
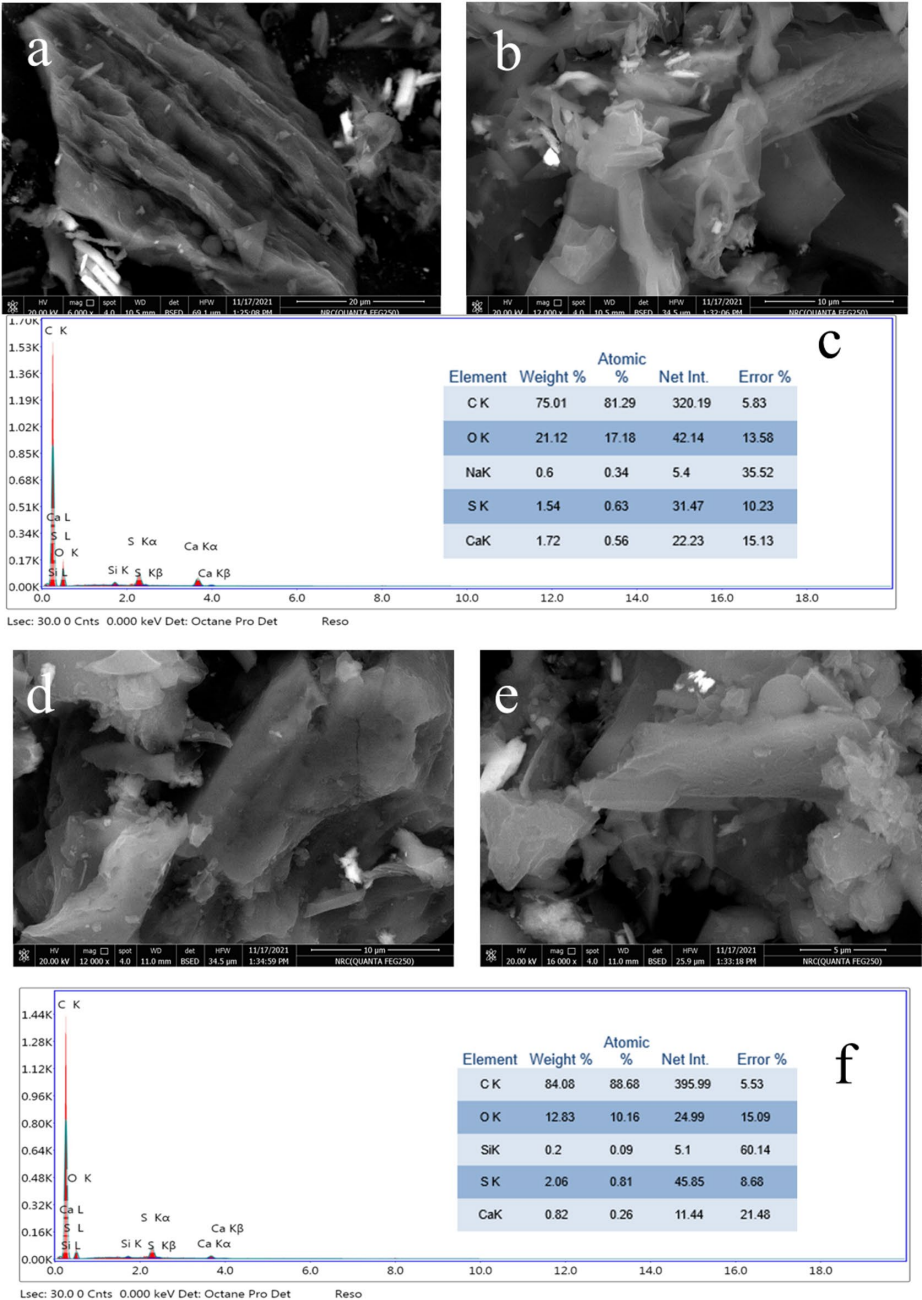
SEM and EDX pictures of MS (**a**–**c**); SEM Image and EDX plot of MS-An@Cs (**d**–**f**).

**Fig. 3:**
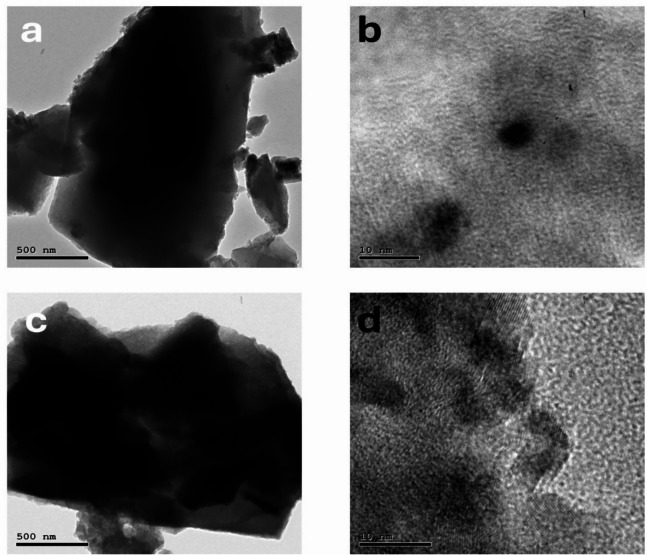
TEM pictures of MS (**a** and **b**) and MS-An@Cs (**c** and **d**).

The morphological characteristics of the MS-An@Cs composite were assessed using SEM-EDX, as depicted in Fig. 3. SEM imaging confirmed the integration of An into MS layers. Figure (2a and b) depicts the clear, unaltered surface of MS, whereas Fig. (2d and e) highlight the emergence of well-formed hexagonal graphite crystals within the composite structure following the addition of An. The EDX spectrum of MS (Fig. 2c) revealed peaks for carbon and oxygen, confirming the structural framework of activated carbon, along with minor silica and calcium traces inherent to MS biomass, were also detected. Upon integrating An with MS (Fig. 2f), the carbon content increased by approximately 10.78%, and the silica content rose from 0.63% to 0.81%. Conversely, the calcium content decreased from 0.56 to 0.26%. These results validate the successful incorporation of An into the MS structure.

**Fig. 4:**
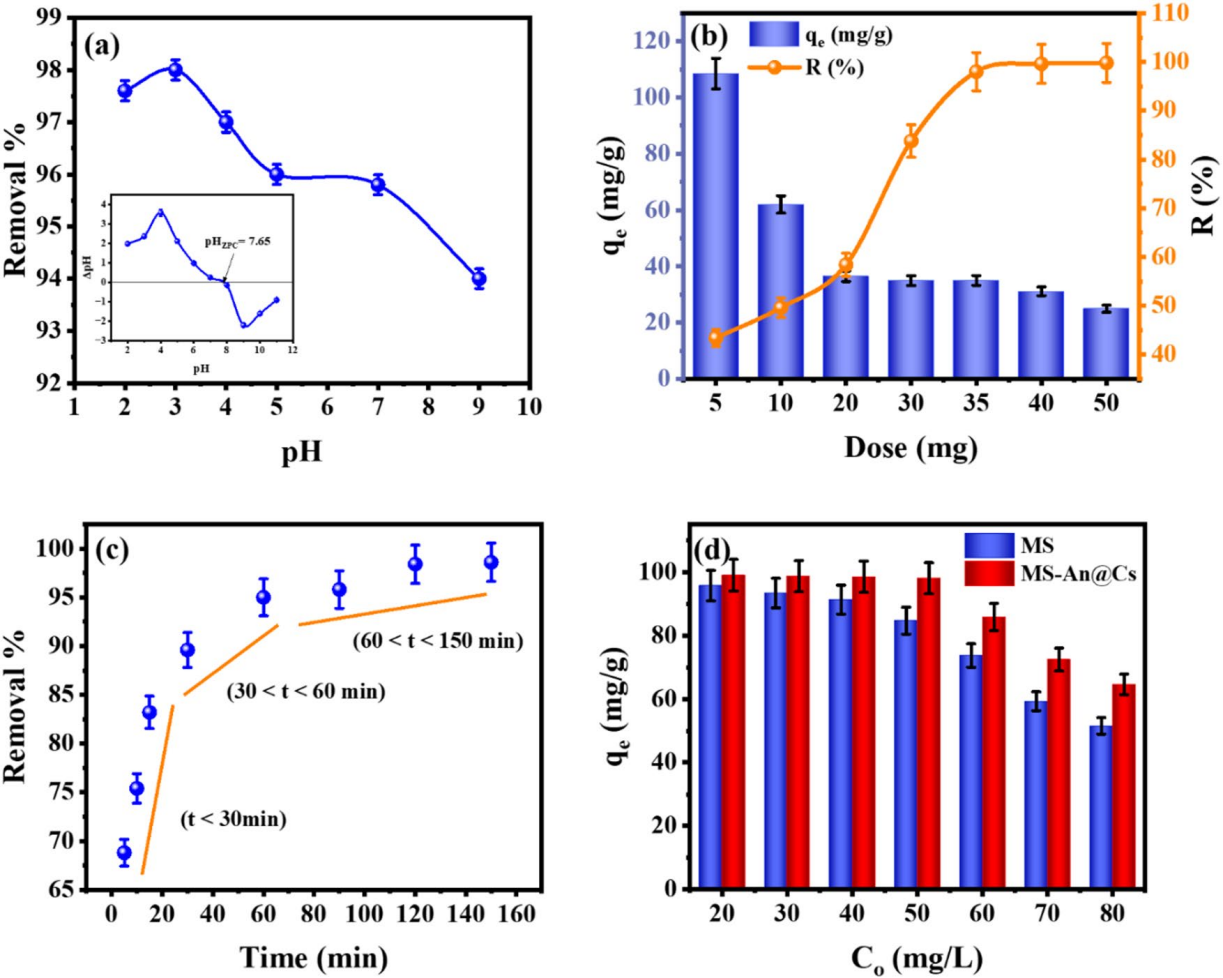
(**a**) Effect of pH (**b**), Effect of adsorbent mass (**c**), Effect of contact time (**d**), and Effect of initial concentration on the MO removal efficiency by MS-An@Cs.

Transmission electron microscopy (TEM) image in Fig. 3c and d shows the interior details of An welding in MS. It is noted that An particles are uniformly welded into the continuous porous network of MS layers, confirming CS reaction can afford a well-developed hybridization between MS and An. Figure 4c exhibits the magnified details of An particles welded by MS and Cs scaffolds in MS-An@Cs. The morphological features of MS-An@Cs in TEM analysis well conform to SEM observations.

### Adsorption study

#### Effect of pH

The role of aqueous pH in modulating MO uptake by the MS-An@Cs adsorbent was examined through experiments spanning acidic to basic conditions (pH 2–9)^42^. All adsorption tests were carried out with a consistent set of variables: 5 mg L⁻¹ initial dye concentration, 35 mg of adsorbent, a temperature of 25 °C, and an equilibration time of 120 min. According to the experimental protocol of^43^, the point of zero charge (pH_PZC_) for the MS-An@Cs adsorbent was identified as 7.65 (Fig. 4a). This parameter signifies the specific pH at which the material’s surface possesses an overall neutral electrical state. The adsorption performance for the anionic MO species was highly sensitive to solution pH, as illustrated in Fig. 4a. The removal percentage decreased from an optimum of 98% at pH 3 to 94% at pH 9^44^. This inverse relationship is a direct consequence of the evolving electrostatic affinity between the dye molecules and the adsorbent surface, which is controlled by the differential between the solution pH and the predetermined pH_PZC_. A net positive surface charge develops at pH levels below 7.65 (the pH_PZC_), largely due to the protonation of amine groups on the chitosan and associated functional moieties^45^. A related consideration is the pKa of MO, which is 3.46. When the ambient pH surpasses this critical point, the MO species predominantly convert to anions, a transformation that significantly favors their adsorption onto positively charged layered structures through Coulombic forces. Consequently, at pH levels below 3.46, the cationic adsorbent surface promotes strong electrostatic interaction with anionic MO species, yielding high sequestration efficiency. In contrast, under alkaline conditions (pH > 7.65), the surface develops a net negative charge, creating electrostatic repulsion that significantly reduces MO adsorption efficency^46^. In summary, the adsorption process is fundamentally controlled by electrostatic interactions. The solution pH serves as a critical regulatory variable, directly manipulating both the surface charge density of the biosorbent and the ionic form of the MO molecules, thereby dictating overall removal efficiency^47^. Research from various studies consistently demonstrates that MO adsorption is optimal at pH = 3^47–50^.

#### Effect of adsorbent amount

Without modifying other parameters, Fig. 4b demonstrates the effect of varying the MS-An@Cs dosage (5–50 mg) on the adsorption of MO. The adsorbent demonstrates a considerable improvement in its removal percentage with the increase in dosage from 5 to 50 mg. The findings from the experiments demonstrate a significant decline in the adsorption capacity (qe) of the adsorbent, falling from 108.5 to 24.95 mg/g. In contrast, there is a notable increase in removal efficiency (%), rising from 43.4 to 99.8%. As the adsorbent dosage increased, the rate of MO removal correspondingly escalated. The observed enhancement in adsorption is mainly due to the increased dosage of the adsorbent, which results in a larger number of adsorption surfaces and sites, ultimately improving efficiency^7^. However, an increase in the adsorbent dosage, with the sorbate concentration held constant, results in a decrease in qe (mg/g). As noted, the MS-An@Cs dosage reached its adsorption saturation point at around 35 mg, with further increases to 50 mg showing no substantial effect on removal efficiency.

The alteration in qe demonstrates that, at a given sorbate concentration (35 mg/25 mL), a higher number of adsorption sites leads to a marked reduction in the adsorption driving force^51^. By evaluating both qe and removal efficiency, it was determined that the optimal and most cost-effective dosage is 35 mg. Thus, the MS-An@Cs dosage of 35 mg was selected for further experimental work.

#### Contact time effect

Contact time plays a vital role in analyzing the equilibrium between the adsorbate and adsorbent. As depicted in (Fig. 4c), the removal efficiency of MO dye starts at 68.8% after 5 min and reaches equilibrium after 120 min, with a final removal of 98.6%. The rapid adsorption of MO was attributed to the increased interaction between the oppositely charged sites on the MS-An@Cs composite. Furthermore, the faster adsorption in the initial stages was due to the ample surface area and the availability of reactive sites on the adsorbent, which became saturated as equilibrium was attained.

#### Initial concentration effect

The impact of varying initial MO concentrations on adsorption performance was explored. As reported in previous studies^52,53^, a concentration range of 20–80 mg/L exerted a considerable influence on the adsorptive performance, while the mass of both MS and MS-An@Cs adsorbents was maintained at a constant quantity of 35 mg per 25 mL. The data presented in Fig. 4d reveal that rising initial concentrations of MO progressively augmented the adsorption capabilities of both materials. MS adsorption grew from 13.67 to 29.46 mg/g, whereas the MS-An@Cs composite exhibited a superior performance, with its capacity surging from 14.143 to 36.93 mg/g. A concomitant reduction in MO removal efficiency was observed as the initial concentration increased. The efficiency for MS fell from 95.7 to 51.56%, while the MS-An@Cs composite maintained a higher yet still declining efficiency, dropping from 99.01 to 64.6%. This phenomenon, wherein capacity increases as fractional removal decreases, corroborates prior research on the competitive dynamics at elevated adsorbate concentrations^32^. This phenomenon is explained by the greater number of MO molecules present at higher concentrations, which promotes increased uptake per gram of the adsorbent material. A parallel finding was reported by Mohamed et al.^7^, who documented a concurrent rise in adsorption capacity and decline in fractional removal during MO adsorption onto carbon nanotube impregnated anthracite.

### Adsorption isotherms

**Fig. 5:**
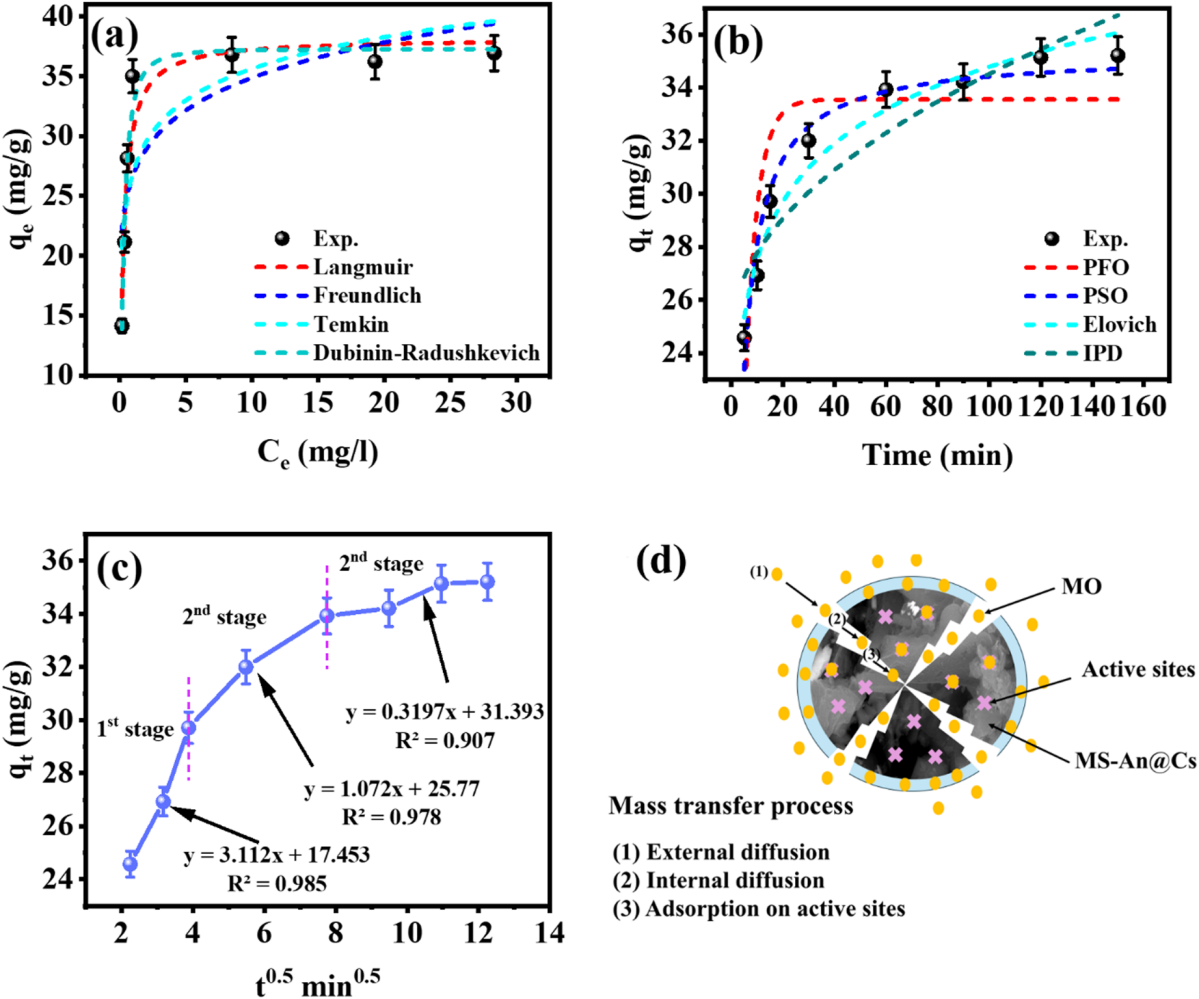
(**a**) adsorption isotherm models, (**b**) adsorption kinetic models, (**c**) IPD model, and (**d**) Schematic diagram of diffusion MO dye onto MS-An@Cs.

In the practical design of adsorption systems, isotherm models are indispensable, serving both to guide adsorbent selection and to facilitate the analysis of equilibrium data^54^. While the current investigation centers on the isotherm characteristics and adsorption efficacy of the MS-An@Cs composite for MO. This investigation characterized the performance of the MS-An@Cs composite through equilibrium experiments performed at 25 °C. The primary aim was to ascertain the equilibrium adsorption capacity (q_e_), defined as the mass of adsorbate retained per unit mass of adsorbent at equilibrium, utilizing the relationship specified in Eq. (2). The equilibrium data for the MS-An@Cs composite, illustrated in Fig. 5, are presented as adsorption isotherms plotting qe against C_e_. The profile of these isotherms yields critical insights into the material’s maximum uptake potential and the affinity of the adsorbent-adsorbate interaction. As illustrated in Fig. 5a, the MS-An@Cs composite achieved a maximum adsorption capacity (qmax) of 36.93 mg/g. This value was determined at a pH of 3, a temperature of 25 °C, a contact time of 2 h, a solution volume of 25 mL, and an initial MO concentration of 80 mg/L. To interpret the resulting equilibrium data, four isotherm models—Langmuir, Freundlich, Temkin, and Dubinin–Radushkevich (D–R)—were employed.

A fundamental distinction between the Langmuir^55^ and Freundlich^56^ isotherm models lies in the conceptualization of the adsorbent surface. The former presumes an energetically uniform surface with no interaction among adsorbed species^32^, whereas the latter accounts for surface heterogeneity^56^. Their respective nonlinear formulations are given in the following Eqs:

4 $$\:{\mathrm{q}}_{\mathrm{e}}={\mathrm{q}}_{\mathrm{m}\mathrm{a}\mathrm{x}}\frac{{\mathrm{K}}_{\mathrm{L}}{\mathrm{C}}_{\mathrm{e}}}{1+{\mathrm{K}}_{\mathrm{L}}{\mathrm{C}}_{\mathrm{e}}}$$

5 $${{\mathrm{q}}_{\mathrm{e}}}={\text{ }}{{\mathrm{K}}_{\mathrm{f}}}{{\mathrm{C}}_{\mathrm{e}}}^{{{\mathrm{1}}/{\mathrm{n}}}}$$

6 $$\:\mathrm{q}\mathrm{e}\:=\:\mathrm{q}\mathrm{m}\mathrm{a}\mathrm{x}\frac{\mathrm{R}\mathrm{T}}{\mathrm{b}}\mathrm{l}\mathrm{n}({\mathrm{K}}_{\mathrm{T}}{\mathrm{C}}_{\mathrm{e}}$$)

7 $$\:{\mathrm{q}}_{\mathrm{e}}={\mathrm{q}}_{\mathrm{m}\mathrm{a}\mathrm{x}}\mathrm{exp}\left(-{\upbeta\:}{{\upepsilon\:}}^{2}\right)$$

8 $$\:{\upepsilon\:}=\mathrm{R}\mathrm{T}\mathrm{l}\mathrm{n}(1+\frac{1}{{\mathrm{C}}_{\mathrm{e}}})$$

9 $$\:{\mathrm{E}}_{\mathrm{D}\mathrm{R}}=\sqrt{\frac{1}{2{\mathrm{K}}_{\mathrm{D}\mathrm{R}}}}$$

In these expressions, Ce signifies the equilibrium concentration of MO in the aqueous phase (mg/L), while qe denotes the MO mass adsorbed per unit mass of MS-An@Cs at equilibrium (mg/g). The parameter qmax represents the theoretical maximum adsorption capacity (mg/g), ε corresponds to the Polanyi potential, and β is a constant associated with the mean free energy of adsorption (mol²/J²). Within these models, q_max_ defines the theoretical maximum for monolayer surface coverage (mg/g), and b is the Langmuir equilibrium constant (L/mg). The Freundlich parameters, k_F_ (mg/g)(L/mg)¹/ⁿ and n, correspond collectively to the adsorbent’s capacity and the favorability of the adsorption process, respectively. The term T denotes the absolute temperature in Kelvin, while R represents the universal gas constant (8.314 × 10⁻³ kJ/mol·K). The parameter bₜ is identified as the Temkin isotherm constant. The calculated parameters and correlation coefficients for the Langmuir, Freundlich, Temkin, and Dubinin–Radushkevich (D–R) isotherm models at 25 °C are compiled in Table 5. The experimental equilibrium data fitted to these models are presented graphically in Fig. 5a^57^.

The experimental adsorption data were evaluated using Langmuir and Freundlich isotherm models, the results of which are graphically presented in Fig. 5a, respectively. Quantitative analysis, summarized in Table 5, demonstrates a stronger correlation with the Langmuir model. This fit indicates a monolayer adsorption mechanism and suggests a high saturation capacity of 38.2 mg/g for the MS-An@Cs adsorbent, a conclusion supported by the model’s elevated R² value and low error functions.

**Table 5:** Adsorption isotherms fitting parameters for MO adsorption onto MS-An@Cs.

Parameter value	Isotherm	Parameter	Error function	Error value
36.93	Langmuir	Q_e_ (exp)	MPSD	4.64
38.2		Q_max_ mg/g	ARE	3.91
3.83		K_L_ L/mg	X^2^	7.48
0.00325–0.0129		R_L_		
0.92		R^2^		
8.5	Freundlich	n	MPSD	1.36
26.6		K_f_ (mg/g)(mg/L)^1/n^	ARE	2.5
0.665		R^2^	X^2^	32.9
1106.1	Temkin	A_T_ L/g	MPSD	16.9
3.83		b_T_ J/mol	ARE	8.98
0.72		R^2^	X^2^	3.67
37.3	DR	q_m_ mg/g	MPSD	4.3
0.31		K_D_ mol^2^/J^2^	ARE	3.7
1.3		E_D_ (kJ/mol)	X^2^	3.84
0.97		R^2^		

As can be seen from Table 6, MS-An@Cs showed a higher adsorption capacity than various adsorbent s that had been previously documented in the literature. Furthermore, it was a viable alternative to adsorbent with comparable or even greater adsorption capacities because of its synthesis from inexpensive and renewable sea grass, which improved its overall efficiency. K_L_, the Langmuir isotherm constant, is correlated with the surface area/pore volume and indicates that a high adsorption capacity would be the outcome of its rise^57^. The separation factor (R_L_) and Freundlich exponent (n) further validated the MO adsorption’s favorability. The adsorbent demonstrated good adsorption when n was greater than unity and R_L_ being 0 < R_L_<1.

Based on the data compiled in Table 5, the Dubinin–Radushkevich (D-R) isotherm provides the most accurate representation of MO adsorption onto the MS-An@Cs composite. The model’s fit reveals an elevated initial adsorption capacity, characterized by a sharp increase in uptake at lower Polanyi potential (ε) values. This trend, which is apparent in both the empirical data and the corresponding power-law regression, signifies enhanced adsorbent efficacy in environments with high solute concentrations. The, characterization of the adsorption interaction, based on the Dubinin-Radushkevich (D-R) isotherm, placed the adsorption energy in the range typical of physical adsorption (< 8 kJ/mol).

**Table 6:** The Opiumium conditions for MO by some natural, modified and synthetic materials in comparison with the MS-An@Cs of the present study.

Adsorbent	Initial MO concentration (mg/L)	Adsorbent mass	pH	qmax (mg/g)	V/M	References
BPCMC-g-poly	50	49 mg/100 ml	7.0	9.470	2.04	^58^
ZIF-8	50	10 mg/ 10 ml	6.50	10.10	1	^59^
BiOClxIy	5	0.2 g	neutral	5.0	0.125	^60^
COMRC	100	0.1 g	2.0	34.48	0.25	^61^
ZSM-5 zeolite	10	0.12 g	1	25	0.5	^62^
ZMAC	100	10 mg	4.0	66.2	2.5	^8^
AC-VVL	100–1000	1 g	2.0	79.7	0.025	^18^
MCM-48/RHA	120–200	0.05 g	3.0	84.307	0.5	^47^
MS-An@Cs	50	35 mg	3	38.2	0.7	This work

Consequently, the predominant mechanism is one of physisorption, driven by van der Waals forces, which contrasts sharply with chemisorption that involves significant chemical bonding^22^. The elevated coefficient of determination (R² = 0.97) for the MS-An@Cs system demonstrates a close correspondence between the empirical data and the power law model, thereby substantiating its applicability for characterizing the adsorption dynamics. Further, ther isotherm modeling indicates that MO adsorption onto the MS-An@Cs composite occurs via a monolayer process, a conclusion supported by the Langmuir model’s superior fit relative to other isotherms. Furthermore, the determined adsorption energy values are consistent with a physisorption mechanism, dominated by weak van der Waals forces instead of chemical bond formation.

### Adsorption kinetics

As shown in Table 7, to further understand the adsorption kinetics of MS-An@Cs, many kinetic models, such as PFO, PSO, Elovich, and IPD^63–66^, were fitted to the experimental data. The PFO model states that the concentration difference between the saturated solute and the solid adsorptive phase over time is exactly proportional to the adsorption rate. The PSO model states that direct interactions between the adsorbent and the adsorbate are mostly responsible for adsorption. The IPD model states that the adsorption process is controlled by the diffusion of adsorbate molecules within the particle to reach active adsorption sites. Conversely, the Elovich model indicates that the rate of adsorption falls exponentially as adsorption rises, most likely due to surface heterogeneity or modifications in the energy distribution.

10 $$\:{\mathrm{P}\mathrm{F}\mathrm{O}:\:\mathrm{q}\mathrm{t}\:=\:\mathrm{q}\mathrm{e}\:(1-\boldsymbol{e}}^{{-\:\boldsymbol{k}}_{1}\boldsymbol{t}}$$)

11 $$\:\mathrm{P}\mathrm{S}\mathrm{O}:\:\mathrm{q}\mathrm{t}\:=\frac{{\boldsymbol{q}}_{\boldsymbol{e}}^{2}{\boldsymbol{K}}_{2}\boldsymbol{t}}{1+{\boldsymbol{q}}_{\boldsymbol{t}}{\boldsymbol{K}}_{2}\boldsymbol{t}}$$

12 $$\:\mathrm{E}\mathrm{l}\mathrm{o}\mathrm{v}\mathrm{i}\mathrm{c}\mathrm{h}\:\mathrm{q}\mathrm{t}=\frac{1}{\boldsymbol{\beta\:}}\boldsymbol{l}\boldsymbol{n}(\boldsymbol{\alpha\:}\boldsymbol{\beta\:}\boldsymbol{t}+1)$$

13 $${\text{IPD }}{{\mathrm{q}}_{\mathrm{t}}}={{\mathrm{k}}_{{\mathrm{diff}}}} \times {{\mathrm{t}}^{0.{\mathrm{5}}}}+{\mathrm{C}}$$

**Table 7:** Adsorption kinetic models parameters.

Model			Parameters			units			value		
PFO			q_e_			mg/g			33.552		
			K_1_			min^− 1^			0.211		
			R^2^						0.753		
			X^2^						0.974		
			RMSE						1.864		
PSO			q_e_			mg/g			35.295		
			K_2_			g.mg^− 1^.min^− 1^			0.011		
			R^2^						0.965		
			X^2^						0.125		
			RMSE						0.704		
Elovich			Α			mg·g⁻¹·min⁻¹			1953.989		
			β			g/mg			0.317		
			R^2^						0.963		
			X^2^						0.137 0.724		
			RMSE								
IPD			K_diff_			mg·g⁻¹·min^1/2^			0.984		
			C			mg/g			24.67		
			R^2^						0.855		
			X^2^						0.559		
			RMSE						1.431		
*Linearized interparticle diffusion*											
Step I				Step II				Step III			
K_diff_	C	R^2^		K_diff_	C		R^2^	K_diff_		C	R^2^
3.112	17.453	0.985		1.072	25.77		0.978	0.3197		31.393	0.907

The fitting results of the nonlinear modeling of the MO dye adsorption rate utilizing the PFO, PSO, Elovich, and IPD equations are displayed in (Fig. 5b). Table 5 summarizes the results of the calculations of all pertinent parameters. The suitability of kinetic models for characterizing the adsorption process was evaluated using the coefficient of determination (R²), chi-square (X²), and root mean square error (RMSE). Statistical analysis identified the pseudo-second-order (PSO) model as the most accurate descriptor of MO adsorption kinetics, evidenced by its superior R² value of 0.965 and the lowest corresponding X² and RMSE values among all models tested.

According to the PSO model, the rate-limiting step is probably chemical sorption or chemisorption^64^, which involves valency interactions between MO and MS-An@Cs through electron sharing or exchange and produces the best correlation with the data that was seen.

Furthermore, the IPD model was fitted to the adsorption data in order to characterize the diffusion process of MB and MO within the MS-An@Cs. Three distinct linear regions can be seen in the adsorption process of MS-An@Cs of MO dye, as illustrated in (Fig. 5c), indicating that a multi-step mechanism governed the adsorption process^65^.

Figure 5d displayed the schematic diagram for the MS-An@Cs adsorption process. The fast adsorption rate was caused by the diffusion of MO molecules from the solution to the MS-An@Cs interface and their quick transfer in the first stage. Internal diffusion dominated the second stage, during which the dye molecules spread further to the free adsorption site in the MS-An@Cs pores. After building up at the adsorption active site, the dye molecules eventually attained saturation in the last stage, producing a constant adsorption capacity.

### Adsorption thermodynamics

An investigation into the adsorption mechanism governing the interaction between methyl orange (MO) and the MS-An@Cs adsorbent was conducted through thermodynamic analysis, with specific focus on the standard state changes in free energy (ΔG°), enthalpy (ΔH°), and entropy (ΔS°). The experiment was set up utilizing a range of temperatures between 288 and 328 K, as indicated in Table 8. The MO adsorption isotherm on adsorbents at different temperatures was used to compute the adsorption thermodynamics parameters.

**Fig. 6:**
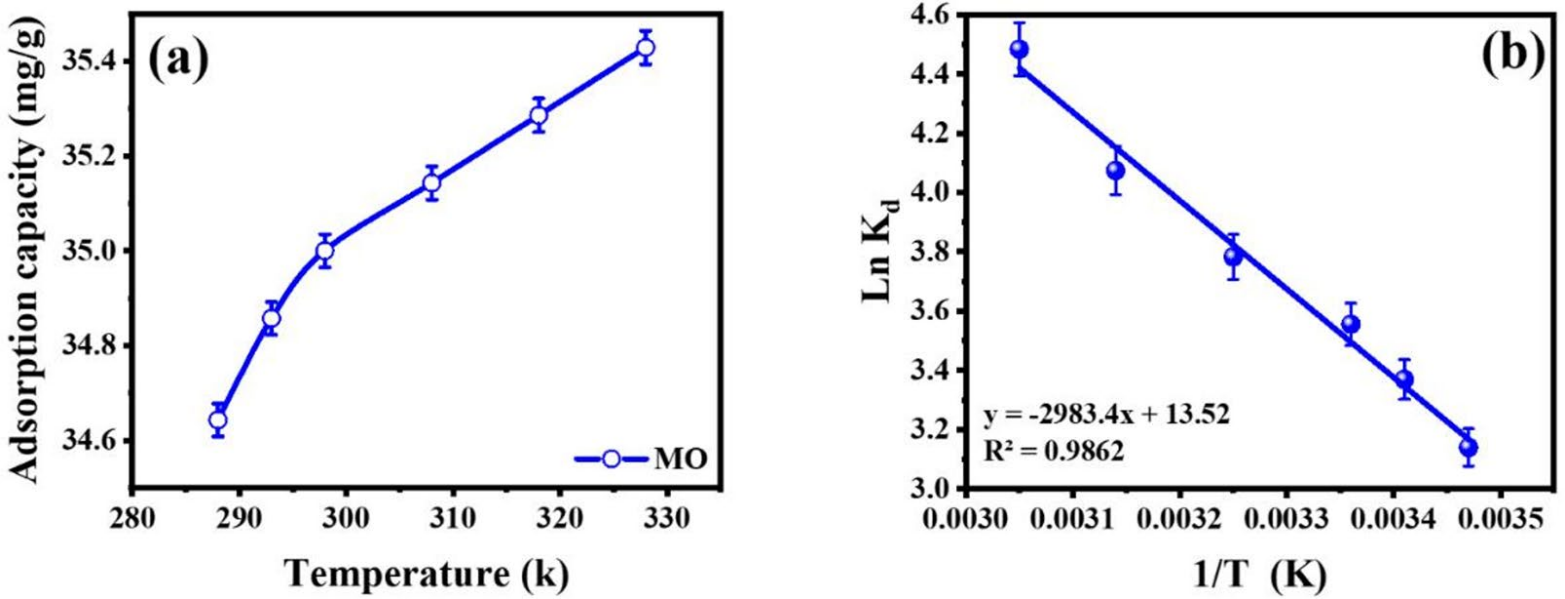
(**a**) Effect of temperature (**a**), Van’t Hoff plot (**b**).

The thermodynamic equilibrium constant (K_d_) is presented as a function of inverse temperature (1/T) in Fig. 6b, with the corresponding parameters compiled in Table 8. The negative values of the ΔG° confirm the spontaneous nature of the adsorption process. Furthermore, the observed decrease in ΔG° magnitude with rising temperature signifies an enhancement in adsorption efficacy at elevated temperatures, which is consistent with an endothermic reaction mechanism^67^. The endothermic nature of the adsorption is corroborated by the positive ΔH° value^68^. For the MS-An@Cs composite, the calculated ΔH° was determined to be 9.368 kJ/mol. An enthalpy change of this magnitude, being below the established 40 kJ/mol threshold, is indicative of a physisorption mechanism. This conclusion regarding the adsorption character aligns with the interpretations derived from the isotherm analysis^69^. Furthermore, the adsorbent and adsorbate have a weak connection, as indicated by the low value of ΔH^o^^70^. Physisorption was indicated by the MS-An@Cs adsorbent’s ΔH^o^ value of 8.270 kJ/mol^71^. The MO adsorption process becomes more random when the ΔS^o^ value is positive. Generally speaking, the degree of irregularity is decreased when dye molecules adsorb onto the adsorbent’s surface. Water molecule desorption occurs during the adsorption process because there are more water molecules desorbed than MO molecules adsorbed^72^. Because MO molecules are bulkier than water molecules, they can adsorb and desorbed water molecules^73^.

**Table 8:** Thermodynamic parameters for the adsorption of MO onto MS-An@Cs.

ΔH^o^ (kJ/mol)	ΔS^o^ (J/(K·mol)	ΔG^o^ (kJ/mol)						*R* ^2^
		288 k	293 k	298 k	308 k	318 k	328 k	
9.368	112.405	−7.52	−8.21	−8.81	−9.69	−10.77	−12.28	0.986

### Utilization MS-An@Cs composite for treatment of real sample

Prior to and after treatment with 10 mg of MS-An@Cs composite, at a pH of 8.1, 25 °C, and 200 RPM agitation speed for two hours.

**Table 9:** The examination of the physicochemical properties of a specific real industrial wastewater sample.

Items	Unit	Results before adsorption with MS-An@Cs	Results after adsorption with MS-An@Cs	*R*%
pH	unitless	8.1	7.1	–
TDS	mg/L	720	750	–
conductivity	µs/cm	1470	1530	–
MO	mg/L	44.2	1.1	97.5

A shown in Table 9, the MS-An@Cs composite shows outstanding performance in real wastewater, selectively removing 97.5% of methyl orange. The minimal changes in TDS and conductivity confirm it targets the dye specifically without altering the overall ionic composition, proving its high potential for practical industrial wastewater treatment applications. whereas the removal percentage of MO reached 89.2, 94, 70, and 93.3, respectively^10,11,13,74^.

#### Proposed adsorption mechanism

The chemical makeup of the adsorbent and adsorbate is necessary to comprehend the particular physicochemical mechanisms involved in the adsorption process^75^. Combinations of electrostatic attraction, hydrogen bonding, π–π stacking, basic-acid interactions, hydrophobic contact, and van der Waals forces are frequently responsible for the adsorption of water contaminants on adsorbents^76^. As a polycyclic aromatic molecule, MO can interact with adsorbents that also contain aromatics through π–π (8–12 kJ/mol), π -cation (8–25 kJ/mol), and π -anion (20–50 kJ/mol)^77^. Because MO contains heteroatoms (atoms of nitrogen, sulfur, and oxygen) on its structure, it can also form weak hydrogen bonds with adsorbents that contain hydrogen species. The most prevalent adsorption process used by various adsorbents to absorb contaminants is electrostatic interaction. With a pKa of 3.4, MO molecules are ionizable, meaning that they can form strong electrostatic contact with adsorbents that are not at the zero charge point. In aqueous solutions, the main drawback is that if the pH is near the adsorbent’s isoelectric point, it cannot be electrically neutral^78^. Chemical adsorption is the main process in the instance of MO removal by MS-An@Cs, which also involves physical adsorption. MO adsorption adhered to the PSO kinetic model, suggesting that the rate-controlling phase is chemisorption. Additionally, physical adsorption occurred, including electrostatic interactions between the negatively charged group (SO3−) on MO and the positively charged surface (H^+^) of MS-An@Cs^79^. The adsorption capacity of MS-An@Cs was increased at low pH levels.

**Fig. 7:**
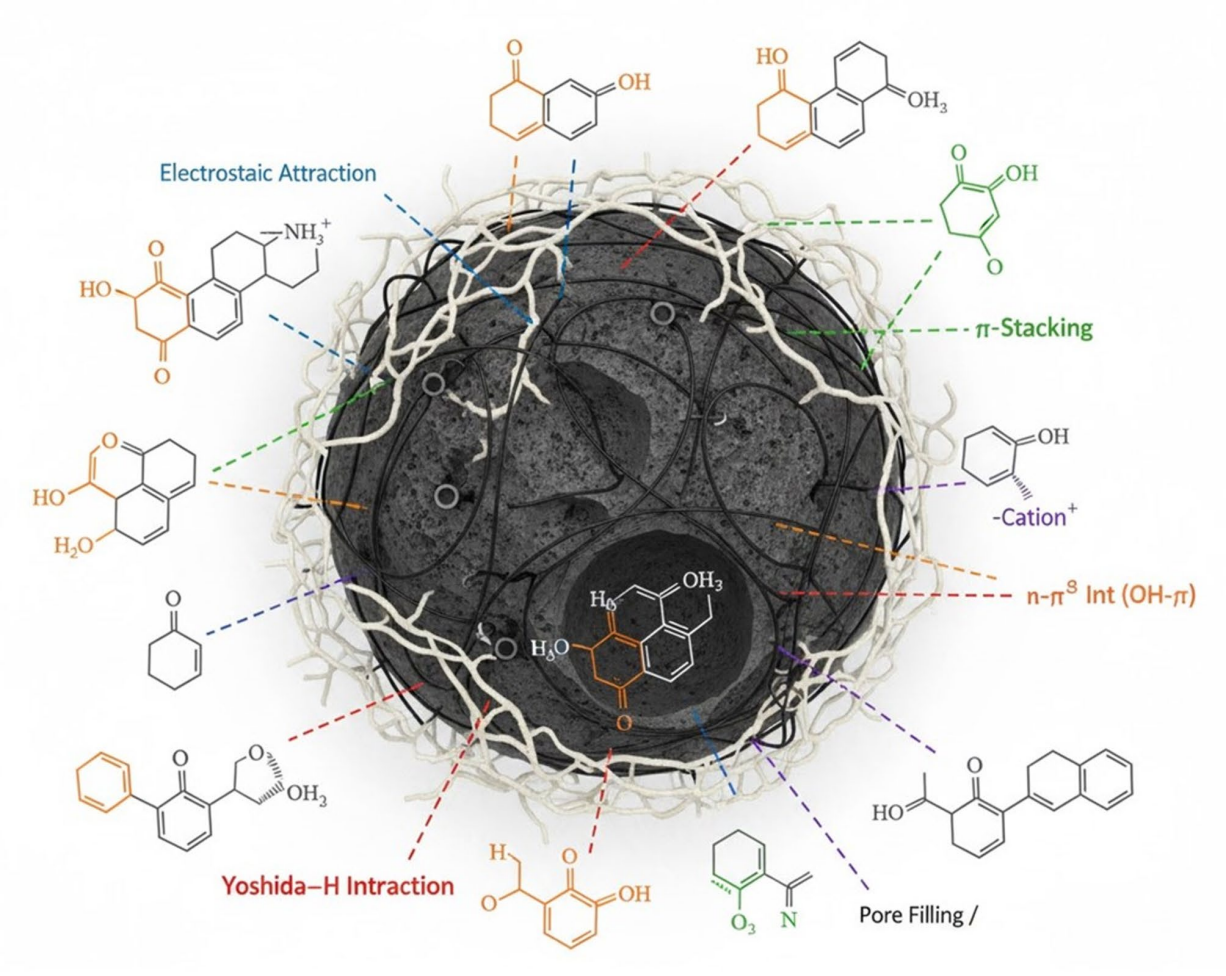
Proposal of the mechanism of MO removal in water by MS-An@Cs.

A negative charge forms when the pH rises and the protonation on the MS-An@Cs surface eventually drops. The negatively charged adsorbent and MO molecules experience electrostatic repulsion, which reduces the adsorption capacity of MS-An@Cs. Additional interactions between MO molecules and MS-An@Cs could occur: On the surface of MS-An@Cs, pore MO may form hydrogen bonds with oxygen-containing functional groups like C–O, C=O, and –OH; π–π interactions between the aromatic rings of the MO dye and the graphitic structure of MS-An@Cs; and an interaction between the H bonds from the OH groups and the delocalized π electron cloud based on the Yoshida-H bonding mechanism^80^. Overall, we can conclude that MO adsorption on MS-An@Cs may be primarily attributed to multiple simultaneous mechanisms, with relative significance based on the conditions below (Fig. 7) and the above mentioned pKa value of MO (i.e., 3.46). These mechanisms include pore diffusion and electrostatic attraction, hydrogen bonding, Yoshida hydrogen bonding, n-π interaction, π-cation interaction, and π-π stacking.

### Regeneration study

**Fig. 8:**
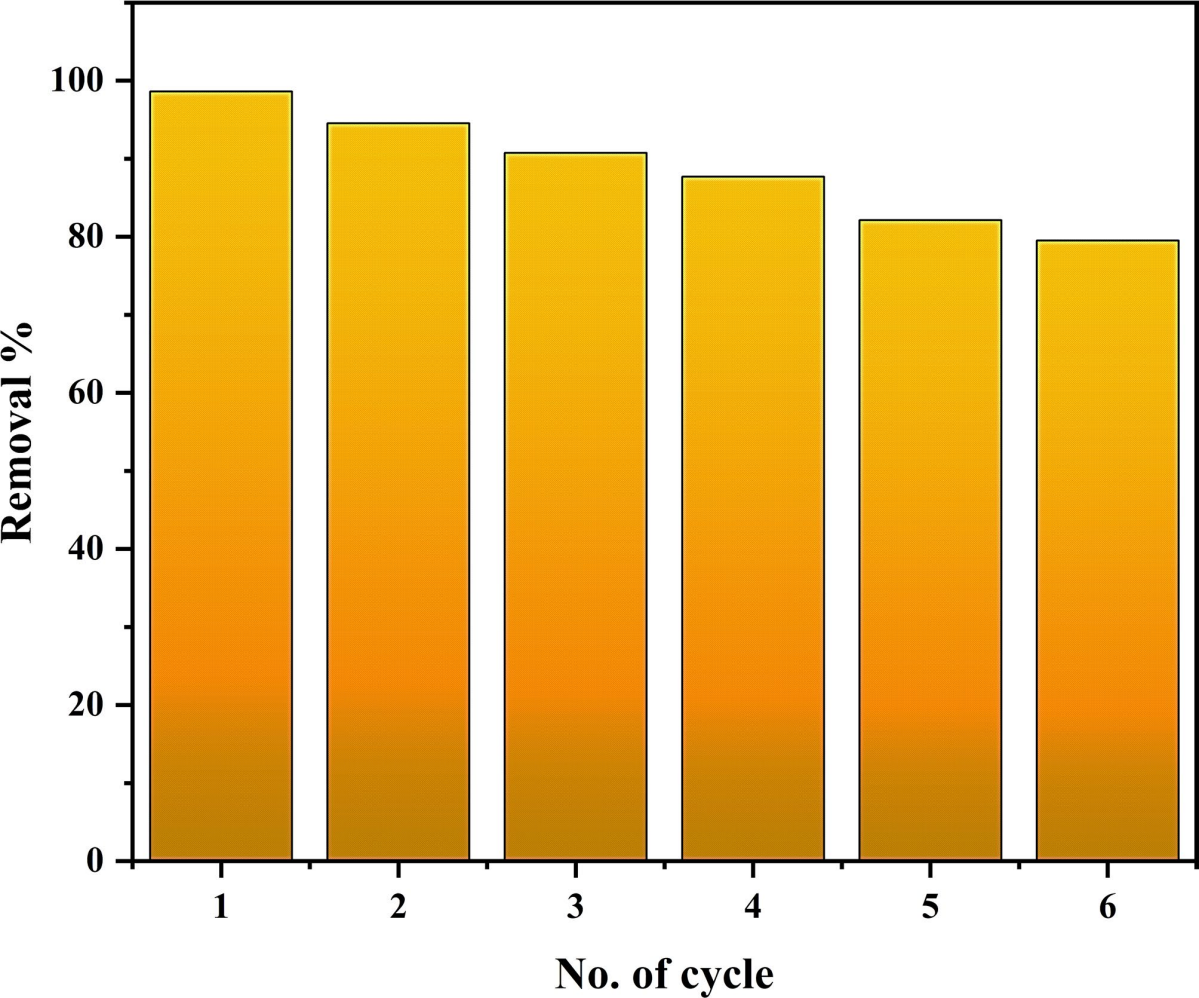
Reusability of MS-An@Cs adsorbent for MO uptake.

One crucial aspect of the adsorption process is the adsorbents’ capacity to be recycled. The utilized adsorbent was centrifuged and stirred in a 0.1 mol/L HCl solution for 90 min to complete the desorption process in this study. It was then rinsed three times in 60 mL of 50% ethanol for 60 min^81,82^. The adsorbent was then taken out and dried so that it could be used again for the subsequent adsorption procedure. The adsorbent’s recyclability performance for up to six reuses is displayed in Fig. 8. After being reused six times, the MS-An@Cs composite’s adsorption performance drops from 98.6 to 79.5%. Because of the MO molecules that remain trapped on the adsorbent surface, new MO molecules cannot attach to the adsorbent, resulting in a decrease in adsorption performance^82^.

### BBD-RSM analysis

ANOVA was employed to evaluate the model’s validity by examining its correspondence with empirical data. As presented in Table 10, the analysis yielded an F-statistic of 256.04 (*p* < 0.0001), demonstrating highly significant model fit and confirming the robustness of the experimental design^23^. Statistical analysis revealed a strong correlation between experimental and model-predicted values, as evidenced by a R² of 0.997. The *p*-value (0.05) was employed to evaluate factor influence on MO removal efficiency. Analysis of variance demonstrated that parameters A (linear term), B (linear term), C (linear term), along with quadratic terms A², and the interaction term AB, AC, BC exhibited statistically significant effects (*p* < 0.05) on the adsorption process. Consequently, nonsignificant variables were excluded to derive the optimized predictive model (Eq. 8) for MO removal.

8 $$\begin{aligned} {\text{MO Removal }}\left( \% \right)= & {\mathrm{72}}.{\mathrm{37}}--{\mathrm{11}}.{\text{72 A}}+{\mathrm{12}}.{\text{79 B}} \\ & +{\mathrm{8}}.{\text{64 C}}+{\mathrm{7}}.{\text{28 AB}}+{\mathrm{5}}.{\text{65 AC}} \\ & +{\mathrm{5}}.{\text{85 BC}}+{\mathrm{12}}.{\text{73 }}{{\mathrm{A}}^{\mathrm{2}}} \\ \end{aligned} $$

**Table 10:** Analysis of variance (ANOVA) for MO removal (%).

Source	SS	df	MS	F-value	*p*-value	
Model	3968.6	9	440.96	256.04	< 0.0001	significant
A-Initial concentration	1098.16	1	1098.16	637.64	< 0.0001	
B-Contact time	1111.33	1	1111.33	645.28	< 0.0001	
C- mass	596.85	1	596.85	346.56	< 0.0001	
AB	211.85	1	211.85	123.01	< 0.0001	
AC	127.46	1	127.46	74.01	< 0.0001	
BC	137.12	1	137.12	79.62	< 0.0001	
A²	682.62	1	682.62	396.36	< 0.0001	
B²	8.7	1	8.7	5.05	0.0594	
C²	4.55	1	4.55	2.64	0.148	
Residual	12.06	7	1.72			
Lack of Fit	4.08	3	1.36	0.6828	0.6074	not significant
Pure Error	7.97	4	1.99			
Cor Total	3980.66	16				

**Fig. 9:**
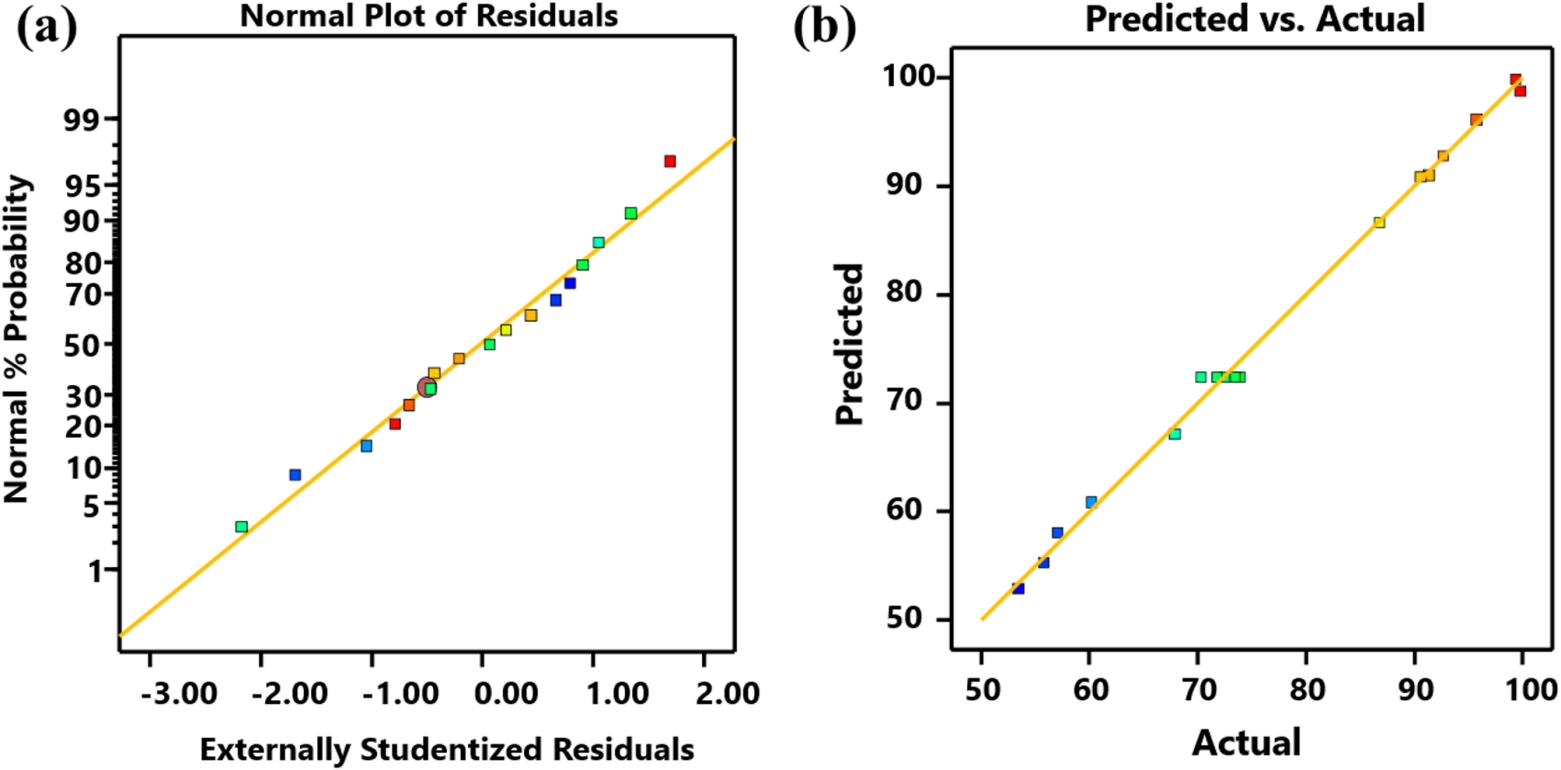
(**a**) Normal probability plot of residuals for MO removal; (**b**) plot of the relationship between the theoretical and real values of MO.

Residual analysis constitutes a critical component in assessing model validity. Figure 9a presents the validation results, demonstrating an appropriate error distribution pattern within the developed model. The observed residuals exhibit uniform dispersion along the reference line, confirming proper model specification and satisfying fundamental regression assumptions. The close correspondence between predicted and experimental values (Fig. 9b) demonstrates strong model reliability, as evidenced by the high degree of concordance in MO removal efficiency measurements^83^. Statistical comparison reveals minimal deviation between theoretical projections and empirical observations, indicating robust predictive capability of the developed model. This quantitative agreement validates the model’s accuracy in simulating the adsorption process. The strong concordance between predicted and observed outcomes substantiates the robustness of the statistical methodology employed, thereby providing compelling empirical validation for the experimental findings^30^.

### Impact of significant interactions on MO removal

**Fig. 10:**
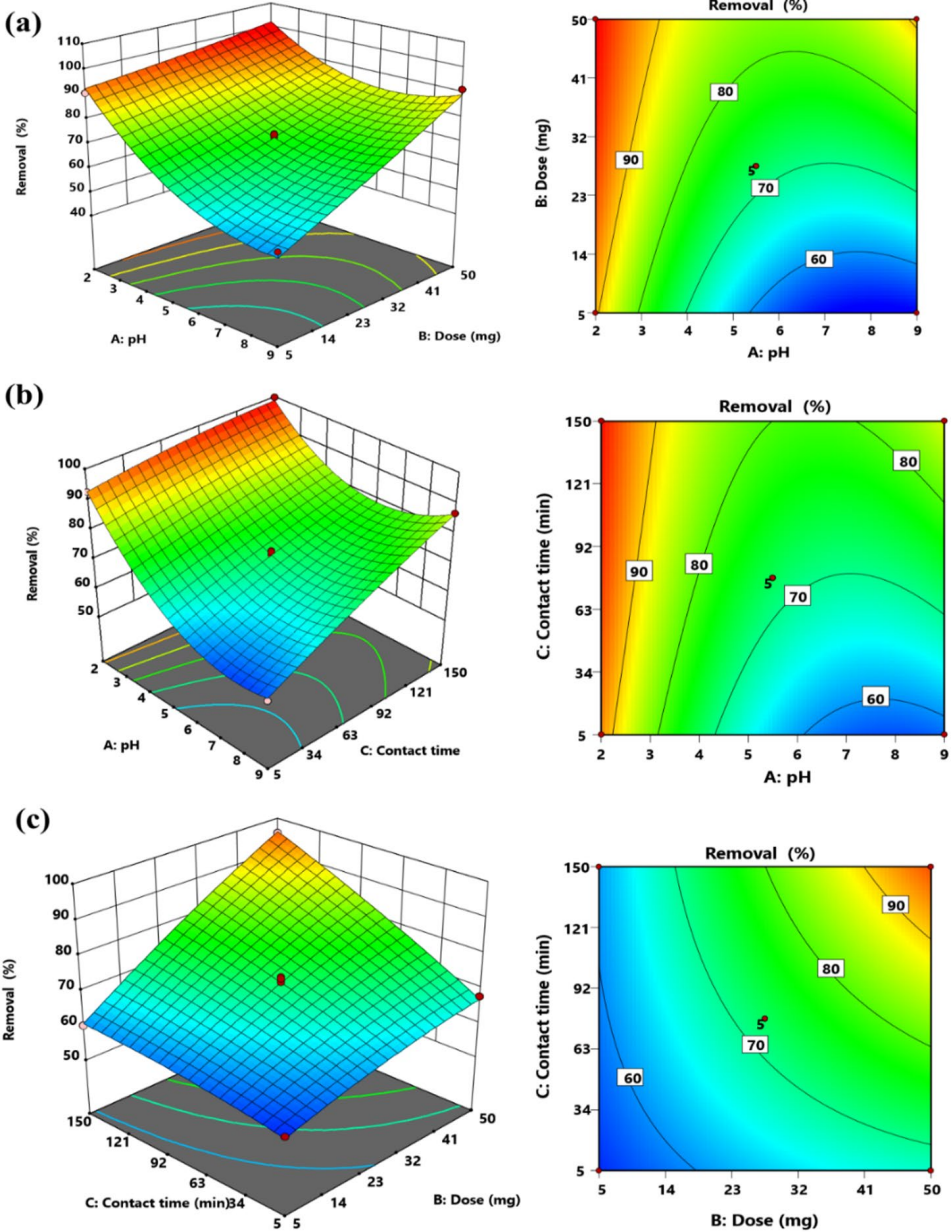
3D response surface plot showing the interaction between (**a**) initial MO mass versus pH, (**b**) contact time versus pH, (**c**) CT versus mass for MO removal.

The interdependencies among key parameters were examined through 3D and 2D contour plots. This investigation elucidates the impact of pH and MS-An@Cs dosage on the MO elimination efficiency, as illustrated in Fig. 10. The graphical analysis reveals an inversely proportional relationship between pH levels and MO removal rates. The removal efficiency of MO exhibited a significant decline as the solution pH increased from 2 to 9. Conversely, elevating the MS-An@Cs dosage from 5 to 50 mg markedly enhanced MO adsorption, as demonstrated in Fig. 10. This improvement can be attributed to the expanded surface area and increased availability of active adsorption sites at higher adsorbent concentrations, facilitating greater contaminant uptake^32^.

Moreover, the experimental data presented in Fig. 9c reveal a pronounced synergistic relationship between MS-An@Cs dosage and contact duration. The adsorption efficiency for MO dye exhibited substantial enhancement with both increasing adsorbent quantity and prolonged exposure time (5–150 min). This phenomenon suggests that extended contact periods coupled with higher MS-An@Cs concentrations facilitate greater occupation of available active sites, thereby improving overall contaminant uptake capacity^30,84^.

#### Cost estimation of the sorptive removal process

The economic viability of adsorbent materials plays a crucial role in determining their suitability for large-scale wastewater treatment applications. The overall manufacturing expense encompasses both energy consumption and raw material costs.

**Table 11:** Cost analysis of the synthesized composite material.

Material	Acquired mass quantity	Total acquisition expenditure (USD)	Unit acquisition cost (USD)	Mass/volume of material consumed	Process material expenditure (USD)
Sea grass	1 kg	–	–	550	–
Anthracite ore	200 g	1	0.005	100	0.5
Chitosan (CS)	50 g	0.5	0.01	10	0.1
Acetic acid	250 mL	0.105	0.00042	10	0.0042
Phosphoric acid	500 mL	1	0.002	250 mL	0.5
Equipment	Time (H)	Rated maximum power (kW)	Specific energy cost (USD/kWh)	Total cost	
Drying	24	1	0.24	5.76	
Calcination	2	1	0.24	0.48	
		Total yield cost = 7.3442 USD For *486.75 g*	Total yield cost 0.015 USD/g		

**Table 12:** Comparative cost analysis of the synthesized MS-An@Cs adsorbent with reported values in the literature.

Material used	Cost (USD/g)		References
Zn-Fe LDH/PANI	12.48	USD/g	^85^
Activated carbon	1.33	USD/kg	^86^
LDH/PU/ O-Pom	0.927	USD/g	^37^
Titania/graphene oxide	0.1875	USD/g	^87^
An/CNTs@CS	0.0326	USD/g	^84^
MS-An@Cs	0.015	USD/g	This study

As detailed in Table 11, the total production cost for 1.0 g of MS-An@Cs composite is estimated at $0.0174. Comparative analysis in Table 12 demonstrates that the MS-An@Cs adsorbent exhibits superior cost efficiency relative to traditional water purification alternatives documented in prior studies.

#### Batch mode scale up design

.

The development of large-scale adsorption systems primarily relies on empirical data obtained from laboratory experiments^32^. Although continuous-operation studies provide the most reliable foundation for scaling adsorption processes, numerous investigators have successfully employed batch-mode experimental findings for industrial-scale implementation^32,83^. This research employs batch adsorption experimental results to upscale the elimination of MO dye via MS-An@Cs nanocomposite under different wastewater treatment capacities. Isotherm modeling demonstrated that the Langmuir model most precisely described the adsorption mechanism. Accordingly, the Langmuir isotherm was incorporated into the mass balance equation to formulate the scaling relationship, as detailed in Eq. (9)^83^.

9 $$\:\frac{W}{V}=\frac{({C}_{0}-{C}_{e})(1+b{C}_{e})}{{C}_{e}{Q}_{m}b}$$

In this context, W represents the weight (gm) of the MS-An@Cs nanocomposite adsorbent, whereas V refers to the total volume (L) of the MO dye solution. The variables C₀ and Cₑ denote the initial and equilibrium concentrations (in mg/L) of the dye, respectively. Furthermore, *b* corresponds to the Langmuir adsorption equilibrium constant, and Qₘ indicates the theoretical maximum adsorption capacity obtained from the Langmuir isotherm analysis.

The necessary amounts of MS-An@Cs nanocomposite for attaining MO dye removal efficiencies between 85 and 95% in wastewater volumes spanning 2.0–50.0 L were calculated using Eq. (9), with the corresponding numerical predictions summarized in Table 13.

**Table 13:** Required dosage of MS-An@Cs nanocomposite for effective MO dye adsorption from aqueous solutions (initial concentration: 50 mg/L) under optimized conditions (temperature: 25 ± 5 °C; pH: 3.0).

	95% removal	90% removal	85% removal	80% removal
Volume of effluents (L)	Mass of MS-An@Cs nanocomposite (g) required for adsorption of MO dye			
2	2.149	1.625	1.102	0.58
4	4.298	3.251	2.204	1.16
6	6.45	4.88	3.305	1.735
8	8.596	6.501	4.407	2.313
10	10.745	8.127	5.511	2.89
20	21.49	16.254	11.02	5.782
30	32.234	24.38	16.53	8.674
40	42.98	32.51	22.04	11.565
50	53.72	40.63	27.55	14.46

The ideal adsorbent quantity for MO dye elimination was determined under consistent experimental conditions, with solution pH, initial MO dye concentration, and contact duration fixed at their pre-established optimal levels. According to the data in Table 13, processing 50 L of effluent with an initial MO concentration of 50 mg/L necessitated 14.46 g, 27.55 g, 40.63 g, and 53.72 g of the MS-An@Cs nanocomposite to attain removal efficiencies of 80%, 85%, 90%, and 95%, respectively. These findings indicate that roughly 53.72 g of the fabricated nanocomposite (Table 13) is sufficient to eliminate 95% of the MO dye from 50 L of contaminated water at the given initial concentration. However, a critical evaluation of the isotherm modeling revealed that the Dubinin-Radushkevich (DR) model provided a superior fit, suggesting it more accurately describes the fundamental adsorption mechanism, likely a pore-filling process. Consequently, while the Langmuir-based calculations offer valuable preliminary scale-up predictions, it is strongly recommended to integrate the DR model into the mass balance for the final, definitive design. This crucial step will enhance the reliability of the scale-up for industrial wastewater treatment by ensuring it reflects the true physical nature of the adsorption.

## Conclusion

This comprehensive investigation successfully establishes the MS-An@Cs composite as a highly efficient and economically viable adsorbent for methyl orange (MO) removal from aqueous solutions. Through systematic optimization, the study identified ideal operational parameters—pH 3, adsorbent dosage of 35 mg, and contact time of 120 min enabling the composite to achieve a maximum monolayer adsorption capacity of 38.2 mg/g. These optimized conditions represent a crucial foundation for practical implementation, ensuring maximum removal efficiency while maintaining operational efficiency.

The mechanistic analysis revealed profound insights into the adsorption process. The excellent fit to the Langmuir isotherm model (R² = 0.93) demonstrates homogeneous monolayer coverage, indicating uniform distribution of active sites on the adsorbent surface. Concurrently, the strong correlation with the pseudo-second-order kinetic model (R² = 0.965) suggests that the rate-limiting step involves physico-sorption-like mechanisms, potentially through electron sharing or exchange between the adsorbent and adsorbate. Further elaboration through intra-particle diffusion modeling uncovered a multi-stage process: initial rapid surface adsorption (0–15 min) followed by gradual pore diffusion (15–60 min), ultimately reaching equilibrium. This understanding of the adsorption kinetics provides valuable information for reactor design and process scaling.

Thermodynamic characterization yielded additional critical evidence regarding the process feasibility. The consistently negative ΔG° values (−7.52 to −12.28 kJ/mol) confirmed the spontaneous nature of MO adsorption, while the positive ΔH° (9.368 kJ/mol) and ΔS° (112.405 J/mol·K) values indicated an endothermic process with increased randomness at the solid-liquid interface during dye removal. Importantly, the ΔH° magnitude firmly established physisorption as the dominant mechanism, with electrostatic interactions playing a pivotal role, particularly at acidic pH conditions where the protonated adsorbent surface strongly attracts anionic MO molecules.

Beyond the fundamental scientific contributions, this research demonstrates remarkable practical applicability. The successful scale-up experiment, where 53.72 g of MS-An@Cs removed 95% of MO from 50 L of contaminated water, validates its potential for real-world implementation. Most significantly, this performance is achieved with exceptional economic efficiency, evidenced by the remarkably low production cost of $0.0174 per gram. This combination of effective contaminant removal and cost-effectiveness positions MS-An@Cs as a superior alternative to conventional water treatment technologies.

## Data Availability

The authors declare that the data supporting the findings of this study are available within the paper.
